# Genomic characterization of patients with polycythemia vera developing resistance to hydroxyurea

**DOI:** 10.1038/s41375-020-0849-2

**Published:** 2020-05-05

**Authors:** Alberto Alvarez-Larrán, Alvaro Díaz-González, Esperanza Such, Elvira Mora, Marcio Andrade-Campos, Carmen García-Hernández, M. Teresa Gómez-Casares, Valentín García-Gutiérrez, Gonzalo Carreño-Tarragona, Marta Garrote, Lierni Fernández-Ibarrondo, José Cervera, Beatriz Bellosillo, Francisco Cervantes, Juan Carlos Hernández-Boluda, On behalf of the MPN Spanish Group (GEMFIN)

**Affiliations:** 1 Hematology Department, Hospital Clínic, Institut d’Investigacions Biomèdiques August Pi i Sunyer (IDIBAPS), Barcelona, Spain; 2 Hematology Department, Hospital La Fe, Valencia, Spain; 3 Hematology Department, Hospital del Mar, IMIM, Barcelona, Spain; 4 Hematology Department, Hospital General Universitario, Alicante, Spain; 5 Hematology Department, Hospital Doctor Negrín, Las Palmas de gran Canaria, Spain; 6 Hematology Department, Hospital Ramón y Cajal, IRYCIS, Madrid, Spain; 7 Hematology Department, Hospital 12 de Octubre, Madrid, Spain; 8 Pathology Department, Hospital del Mar, IMIM, Barcelona, Spain; 9 Hematology Department, Hospital Clínico, INCLIVA, Valencia, Spain

**Keywords:** Myeloproliferative disease, Cancer genetics

## To the Editor:

Resistance/intolerance to hydroxyurea (HU) develops in 20–30% of patients with polycythemia vera (PV) and has been associated with increased risk of thrombosis, disease transformation and lower survival [1–5]. Recently, a molecular classification of myeloproliferative neoplasms (MPNs) based on data provided by next-generation sequencing (NGS) techniques has been proposed [6]. This classification, which stratifies MPN patients into eight molecular groups, may potentially be useful for personalizing prognosis and treatment; however, the impact of this genomic classification in the risk of development of HU resistance is unknown.

The objective of the present study was to characterize PV patients with resistance to HU by genomic classification and to assess its value in predicting disease progression and survival. Samples obtained from 61 HU-resistant patients at diagnosis (*n* = 38) and/or at time of resistance (*n* = 45) were analyzed by NGS. Cases with paired samples (*n* = 22) were sequenced at both time points. Fifty-nine HU-treated patients with no developed resistance were also sequenced by NGS and used as controls. Patients were hierarchically allocated into eight molecular subgroups according to the algorithm reported by Grinfeld et al. [6]. Patients were classified according to the result of the available sample or at time of resistance in those with paired samples. Resistance to HU was assessed according to the ELN modified criteria as described by Barosi et al. [7] and PV diagnosis was established according to World Health Organization criteria [8]. Informed consent was obtained for scientific use of the patients’ clinico-hematological data and biological samples, and the study was approved by the Instituto de Investigación Sanitaria La Fe institutional review board. Survival analyses and time-to-event curves were calculated from date of HU initiation.

**Fig. 1:**
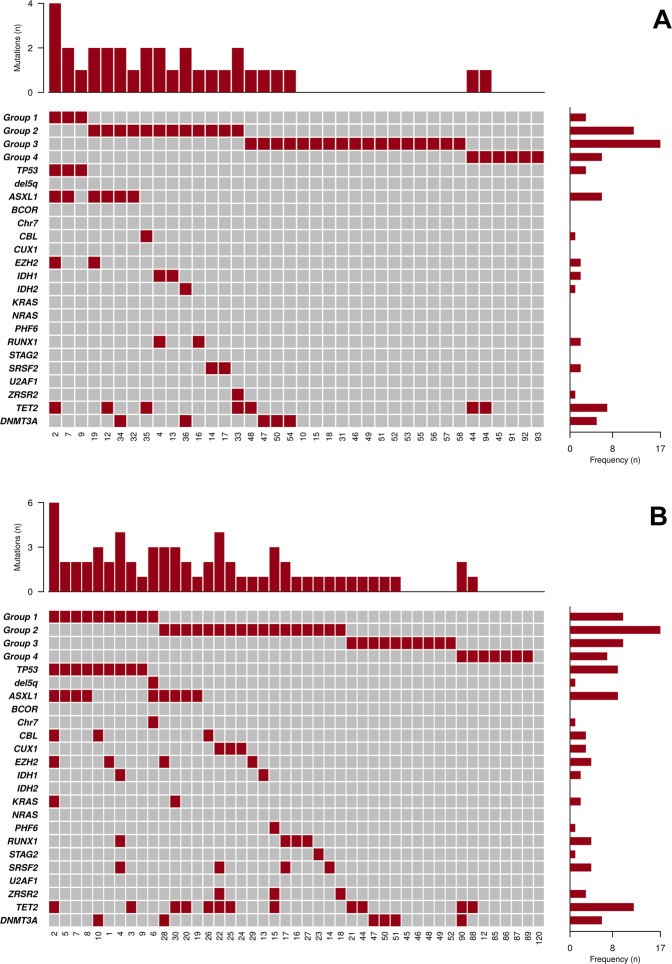
Distribution of mutations other than *JAK2* detected by NGS in 61 hydroxyurea-resistant patients. NGS studies were performed at diagnosis (**a**) or at resistance (**b**). Group 1: *TP53* disruption or aneuploidy (*TP53* mutation, Chr17pLOH or Chr5-/Chr5q-). Group 2: ≥1 aberrations in chromatin or spliceosome genes (*EZH2*, *IDH1*, *IDH2*, *ASXL1*, *PHF6*, *CUX1*, *ZRSR2*, *SRSF2*, *U2AF1*, *KRAS*, *NRAS*, *GNAS, CBL*, Chr7/7qLOH, Chr4q/LOH, *RUNX1*, *STAG2*, and *BCOR*). Group 3: *JAK2* homozygous mutation. Group 4: *JAK2* heterozygous mutation. In the central panel, each column corresponds to an individual case and colored squares denote mutated genes. The upper graph represents the number of mutations in each patient. The right graph illustrates frequency of mutated genes. All patients except one were *JAK2*-positive. No mutation was detected in patient number 120.

The main clinical characteristics of the 61 PV patients with resistance to HU and the 59 controls are shown in supplemental Table 1. Median time on HU therapy was 4.6 years in cases and 5.9 years in controls (p not significant for the comparison). Types of resistance were need for phlebotomies (*n* = 13), progressive splenomegaly (*n* = 3), uncontrolled myeloproliferation (*n* = 6), and cytopenia (*n* = 39). All patients except one were *JAK2*-positive. Mutations other than *JAK2* were frequently observed in resistant patients, which were reclassified as PV with *TP53* disruption/aneuploidy (16.4%), PV with spliceosome/chromatin mutations (37.7%), PV with homozygous *JAK2* mutation (27.9%), and PV with heterozygous *JAK2* mutation (16.4%), whereas the corresponding figures in controls were 1.7%, 11.9%, 44%, and 41%, respectively (*p* < 0.0001, Supplemental Table 2). Distribution of mutations by genomic classification in HU-resistant patients is shown in Fig. 1. In resistant patients with paired samples at diagnosis and at time of resistance, a total of 18 new variants were documented in 9 patients corresponding to a rate of 12.9 new mutations × 100 person years. The main characteristics of these nine patients are shown in supplemental Table 3. The genomic subgroup changed at the time of HU resistance in six cases (27%), four of them as a result of mutation acquisition in *TP53* or *ZRSR2* genes (two cases each) and the remaining two due to transition from heterozygous to homozygous *JAK2* mutational status.

**Fig. 2:**
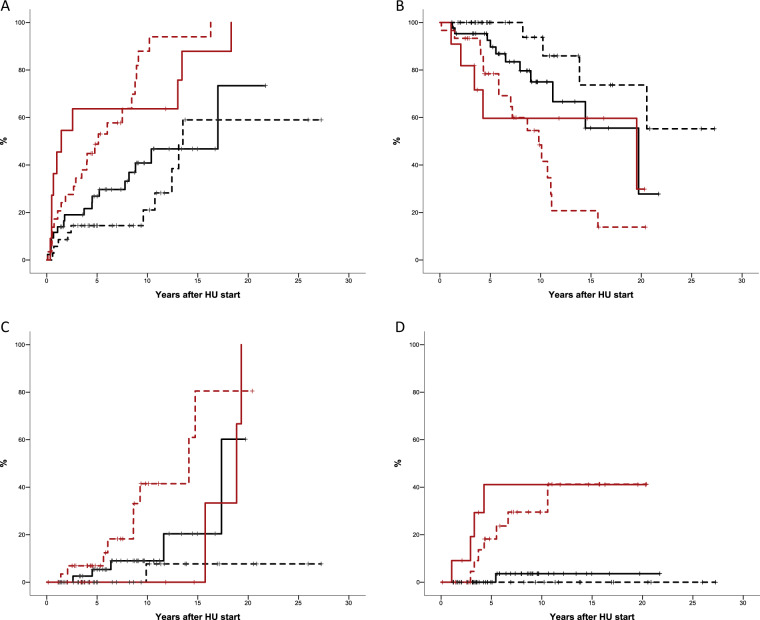
Outcomes according to genomic classification in 120 patients with polycythemia vera treated with hydroxyurea (HU). **a** Time to hydroxyurea resistance. **b** Probability of survival. **c** Probability of myelofibrotic transformation. **d** Probability of progression to acute myeloid leukemia. Solid red line: *TP53* disruption/aneuploidy. Dotted red line: spliceosome/chromatin aberration. Solid black line: homozygous *JAK2* mutation. Doted black line: heterozygous *JAK2* mutation. *p* < 0.0001 for all comparisons.

Time to HU resistance according to genomic classification is shown in Fig. 2a. The probability of developing resistance after 5 years of HU treatment was 64% in patients with *TP53* disruption/aneuploidy, 49% with spliceosome or chromatin aberrations, 27% with homozygous *JAK2* mutation, and 14.5% with heterozygous *JAK2* mutation (*p* < 0.0001 for comparison between groups). In multivariate analysis, genomic classification was associated with risk of resistance to HU (HR 2.2, 95% CI: 1.5–3.2, *p* < 0.0001) after correction for age (HR: 1.01, 95% CI: 0.97–1.03, *p* = 0.9), sex (HR: 0.7, 95% CI: 0.9–1.2, *p* = 0.4), Hb value (HR: 1.08, 95% CI: 0.9–1.2, *p* = 0.3), leukocyte count (HR: 1.1; 95% CI: 1.01–1.18, *p* = 0.02), platelet count (HR: 1.0, 95% CI: 0.9–1.002, *p* = 0.9), and spleen size (HR 1.03, 95% CI: 0.9–1.2, *p* = 0.7). Type of resistance according to molecular subgroup is shown in Supplemental Table 4.

With a median follow-up of 7 years from HU initiation (6.5 years for cases and 7.3 for controls, *p* = 0.6) 38 patients died, resulting in a projected median survival of 15.7 years from HU initiation (95% CI: 7.6–23.8). Probability of survival at 10 years from HU initiation was 94%, 75%, 48%, and 59% in patients with heterozygous *JAK2* mutation, homozygous *JAK2* mutation, aberrations in spliceosome/chromatin genes, and *TP53* disruption/aneuploidy, respectively (Fig. 2b, *p* < 0.0001). In multivariate analysis and after correction for age, patients with *TP53* disruption/aneuploidy or spliceosome/chromatin mutations showed increased risk of death compared with *JAK2* heterozygous patients (HR: 4.2, 95% CI: 1.2–15.1, *p* = 0.026) and *JAK2* homozygous patients (HR: 2.1, 95% CI: 1.01–4.04, *p* = 0.046).

There were no significant differences in thrombosis incidence by genomic classification except between *JAK2* homozygous and heterozygous patients (*p* = 0.047). Patients with spliceosome or chromatin gene aberrations progressed faster to MF than those in the other molecular groups whereas the rate of progression to MF was similar among patients with *TP53* disruption/aneuploidy and *JAK2* homozygosity (Fig. 2c). The probability of MF remained low only in *JAK2* heterozygous patients (*p* < 0.0001, Fig. 2c). Nine progressions to AML and three to myelodysplastic syndrome (MDS) were registered mainly in patients with *TP53* disruption/aneuploidy or aberrations in spliceosome/chromatin genes (*p* < 0.0001, Fig. 2d).

To the best of our knowledge, this is the largest study addressing the genomic complexity of PV patients developing resistance to HU and the first to apply the recently proposed MPN molecular classification in this clinical setting [6]. As expected, resistant patients were frequently located in the high-risk molecular groups, which would explain the high rate of disease progression and worse survival reported in such patients [1, 2]. Thus, 17% of HU-resistant patients corresponded to the *TP53* disruption/aneuploidy category, while up to 40% were classified within the group with spliceosome/chromatin gene mutations, percentages clearly higher than the ones observed in non-resistant HU-treated controls and general PV patients [6]. Moreover, in up to 27% of patients the molecular group allocated at diagnosis had changed at time of resistance, highlighting the importance of sequential studies in patients receiving cytoreductive treatment. The latter finding could be explained by the existence of subclonal mutations not detectable at diagnosis that would expand under HU therapy, or by the appearance of new mutations due to genomic instability [9–11].

HU resistance developed especially rapid in patients with *TP53* mutation. Similarly, a high proportion of patients with mutations in spliceosome/chromatin genes developed resistance to HU, but in this case the number increased progressively during follow-up, affecting virtually all patients at longest follow-up. Molecular classification revealed a particular pattern of progression under HU therapy in each molecular subgroup. Thus, in patients with *TP53* disruption the natural history of the disease was affected mainly by the high probability of AML in the first five years of therapy, while in patients with mutations in spliceosome/chromatin genes, transformation to MF was more notable. High rates of thrombosis and progression to MF were observed in the *JAK2* homozygous mutation group. Finally, patients classified as heterozygous *JAK2* mutation had low rates of resistance, thrombosis and disease progression during HU treatment.

The main limitation of the present study is the relatively small number of patients included and the lack of samples from PV diagnosis in a proportion of cases. However, it is important to underline that there were no significant differences between cases and controls regarding general characteristics such as age, sex, history of thrombosis or duration of HU treatment, and that the distribution of molecular groups in the controls was totally superimposable to that described in Grinfeld et al.’s pivotal study [6].

In conclusion, our data should prove useful in clinical decision-making. They support the implementation of NGS techniques in clinical practice to select the most appropriate treatment for PV patients. New drugs are clearly needed for PV patients with high molecular risk, since results of HU treatment are poor. Improvement of preventive measures for thrombosis is essential in HU-treated patients with homozygous *JAK2* mutation. Conversely, patients classified within the heterozygous *JAK2* mutation category can benefit from long-term cytoreduction with HU, as demonstrated by their low rates of resistance, thrombosis and disease progression.

## Supplementary information

Supplemental material
