# Supplementary material for: Genomic characterization of patients with polycythemia vera developing resistance to hydroxyurea
Source: Leukemia. 2020 May 5;35(2):623–7. doi: 10.1038/s41375-020-0849-2 (PMC7862052; doi:10.1038/s41375-020-0849-2)
Supplement: Supplementary file 1 — Supplemental material [file 41375_2020_849_MOESM1_ESM.docx]

**Supplemental material** **materials contain: supplementary methods and 4 supplementary tables.**

*Supplemental methods*

Resistance to HU was assessed according to the ELN modified criteria as described by Barosi et al: need for phlebotomy to keep hematocrit < 0.45 L/L after 3 months of the maximum tolerated dose (MTD) of HU; uncontrolled myeloproliferation (platelet counts > 400 x 10^9^/L and WBC counts > 10 x 10^9^/L after 3 months of HU MTD); failure to reduce massive splenomegaly (> 10 cm from the costal margin) by more than 50% as measured by palpation or failure to completely relieve splenomegaly-related symptoms after 3 months of HU MTD; absolute neutrophil count < 1 x 10^9^/L or Hb < 100 g/L or platelet counts < 100 x 10^9^/L at the lowest dose of HU required to achieve a complete or a partial response.

Targeted sequencing using a commercial panel (Panmyeloid Solution panel by Sophia Genetics®) enabled accurate and comprehensive detection of SNVs, Indels and CNVs in 63 genes for myeloid neoplasms (S1). The panel included the coding regions of the following genes: *ANKRD26, ASXL1, ATRX, BCOR, BCORL1, CALR, CBL, CDC25B, CEBPA, CSF3R, CSNK1A1, CUX1, DDX41, DNMT3A, EGFR, EGR1, ETNK1, ETV6, EYA1, EZH2, FGFR1, FLT3, GATA1, GATA2, IDH1, IDH2, IKZF1, JAG1, JAK2, KIT, KMT2A, KRAS, LYN, MEF2C, MPL, MTRR, MYBL2, MYC, NF1, NPM1, NRAS, PHF6, PPM1D, PTPN11, PTPRT, RAD21, RUNX1, SETBP1, SF3B1, SH2B3, SMC1A, SMC3, SRP72, SRSF2, STAG2, TAS2R1, TES, TET2, TP53, U2AF1, WRN, WT1, ZRSR2*. The CNVs allowed to detect the main cytogenetic alterations in PV *[del(5q): EGFR, CSNK1A1, DDX41, NPM1* and *TAS2R1; del(7q) CUX1, TES, EZH2* and *IKZF; del(20q): ASXL1, MYBL2, PTPRT* and *JAG1; [+8: FGFR1, MYC* and *RAD21; del(17p): TP53, NF1, SRSF2* and *PPM1D].* This panel was performed on Illumina MiSeq and allowed detection rate of ≥ 99% of target region with coverage ≥ 1000x. The results was analyzed by SOPHiA DDM© platform. All selected variants were categorized into benign, likely benign, variant of uncertain significance (VUS), likely pathogenic or pathogenic by following the “Standards and guidelines for the interpretation of sequence variants” from the American College of Medical Genetics and Genomics and the recommendations to filtering of variants to identify high confidence somatic calls proposed by Grinfeld et al (6). Genomic classification was performed as reported by Grinfeld et al. (6). Briefly, patients were hierarchically allocated into eight molecular subgroups: *TP53* disruption or aneuploidy (*TP53* mutation, Chr17pLOH or Chr5-/Chr5q-); ≥1 genetic aberrations in chromatin or spliceosome genes (*EZH2*, *IDH1*, *IDH2*, *ASXL1*, *PHF6*, *CUX1*, *ZRSR2*, *SRSF2*, *U2AF1*, *KRAS*, *NRAS*, *GNAS,* *CBL*, Chr7/7qLOH, Chr4q/LOH, *RUNX1*, *STAG2* and *BCOR*); *CALR* mutation; *MPL* mutation; homozygous *JAK2* mutation; heterozygous *JAK2* mutation; myeloid neoplasm with other clonal driver mutation, and myeloid neoplasm with no known driver mutation.

Overall survival and time-to-event curves were drawn using the method of Kaplan-Meier with log-rank test for comparisons. Since patient selection was based on having received HU, survival analyses and time-to-event curves were calculated from date of HU initiation. Event-free survival under HU was calculated as time to HU resistance, end of HU therapy, development of thrombosis or disease progression, whichever occurred first. Variables evaluated for their potential prognostic significance were age, sex, history of thrombosis, hematological values at diagnosis, resistance to HU and genomic classification. Multivariate analyses of the factors predicting resistance to HU, survival or hematologic transformation were done by Cox regression. In multivariate analysis including molecular classification, heterozygous *JAK2* mutation was considered the reference group and coded as 0. The other genomic subgroups, including homozygous *JAK2* mutation, chromatin/spliceosome aberrations and TP53 mutation, were coded as 1, 2 and 3, respectively. All statistical analyses were performed with SPSS, version 23.

*Supplemental results*

| **Supplemental Table 1. Main clinical and hematological characteristics at diagnosis in 120 patients with polycythemia vera according to development of hydroxyurea resistance (cases) or not (controls)** | | | |
| --- | --- | --- | --- |
|  | **Cases**  N=61 | **Controls**  N=59 | **P value** |
| Age, years* | 66 (36-88) | 66 (20-84) | 0.3 |
| Female, n (%) | 26 (43) | 25 (42) | 0.9 |
| History of thrombosis, n (%) | 20 (33) | 16 (27) | 0.5 |
| Hemoglobin, g/L* | 179 (145-223) | 170 (146-238) | 0.1 |
| Leukocyte count x10^9^/L* | 12.3 (5.3-26.6) | 11.1 (4.8-20.4) | 0.03 |
| Platelet count x10^9^/L* | 508 (123-1302) | 534 (163-974) | 0.6 |
| *JAK2* mutation  V617F, n (%)  Exon 12, n (%) | 59 (96.7)  1 (1.6) | 59 (100) | ns |

| **Supplemental table 2. Genomic classification of patients with polycythemia developing hydroxyurea resistance (cases) or not (controls)** | | |
| --- | --- | --- |
| **Molecular Group, n (%)** | **Cases**  N=61 | **Controls**  N=59 |
| TP53 disruption or aneuploidy | 10 (16.4) | 1 (1.7) |
| Chromatin or spliceosome gene mutation | 23 (37.7) | 7 (11.9) |
| Homozygous *JAK2* mutation | 17 (27.9) | 26 (44.1) |
| Heterozygous *JAK2* mutation | 10 (16.4) | 25 (42.4) |
| Other driver mutation | - | - |
| No mutation | 1 (1.6) | 0 |

Patients were hierarchically classified according to the algorithm described by Grinfeld et al. P < 0.0001 for comparison.

| **Supplemental table 3. Main clinical and molecular features in 9 PV patients with acquisition of new mutations at time of hydroxyurea resistance** | | | | | |
| --- | --- | --- | --- | --- | --- |
| **Case** | **Age/sex** | **At diagnosis**  **(Gene: VAF)** | **At resistance**  **(Gene: VAF)** | **Molecular group** | **Outcome** |
| 2 | 78/M | *JAK2*: 96%  *TP53*: 5%  *ASXL1*: 51%  *EZH2*: 46%  *TET2*: 48%  *PTPN11*: 28% | *JAK2*: 91%  *TP53*: 5%  *ASXL1*: 53%  *EZH2*: 38%  *TET2*: 46%  *PTPN11*: 25%  ***CBL*: 31%**  ***KRAS*: 3%**  ***SH2B3*: 1%** | TP53 disruption | Cytopenia at 0.5 y  Dead |
| 4 | 68/M | *JAK2*: 77%  *IDH1*: 49%  *RUNX1*: 4% | *JAK2*: 69%  *IDH1*: 49%  *RUNX1*: 46%  ***TP53*: 57%**  ***SRSF2*: 45%** | **Diagnosis:**  Spliceosome/  chromatin **Resistance:**  TP53 disruption | Cytopenia at 0.6 y  AML at 1.1 y  Dead |
| 7 | 79/F | *JAK2*: 100%  *TP53*: 4%  *ASXL1*: 10% | *JAK2*: 67%  *TP53*: 32%  *ASXL1*: 26%  ***PTPN11*: 16%** | TP53 disruption | Cytopenia at 2.5 y  AML at 4.3 y  Dead |
| 10 | 69/F | *JAK2*: 74% | *JAK2*: 74%  ***TP53*: 32%**  ***SH2B3*: 8%**  ***CBL*: 1%**  ***DNMT3A*: 1%** | **Diagnosis:**  *JAK2* homo  **Resistance:** TP53 disruption | Cytopenia at 18 y  MF at 19 y  Alive + 20 y |
| 15 | 83/M | *JAK2*V617F: 94% | *JAK2*: 39%  ***ZRSR2*: 63%**  ***PHF6*: 3%**  ***TET2*: 25%**  ***TET2*: 28%** | **Diagnosis:**  *JAK2* homo  **Resistance:**  Spliceosome/  chromatin | Cytopenia at 2.8 y  MDS at 5.5 y  Dead |
| 16 | 76/F | *JAK2*: 5%  *RUNX1*: 3% | *JAK2*: 11%  *RUNX1*: 2%  ***SETBP1*: 20%** | Spliceosome/  chromatin | Cytopenia at 3.5 y  AML at 3.7 y  Dead |
| 17 | 69/M | *JAK2*: 34%  *SRSF2*: 5% | *JAK2*: 2%  *SRSF2*: 8%  ***TET2*: 14%** | Spliceosome/  chromatin | Cytopenia at 3.9 y  AML at 4.3 y  Dead |
| 18 | 64/M | *JAK2*: 94% | *JAK2*: 97%  ***ZRSR2*: 81%** | **Diagnosis:**  *JAK2* homo **Resistance:**  Spliceosome/  chromatin | Cytopenia at 9.1 y  MF at 9.2 y  Alive + 10 y |
| 51 | 56/M | *JAK2*: 70% | *JAK2*: 94%  ***DNMT3A*: 50%** | *JAK2* homo | Cytopenia at 10.4 y  MF at 11.6 y  Alive + 14 y |

New mutations detected at time of resistance are highlighted in bold. F: female. M: male. MDS: myelodysplastic syndrome. AML: acute myeloid leukemia. MF: secondary myelofibrosis. Y: years. VAF: variant allele frequency. Patients were hierarchically classified according to the algorithm described by Grinfeld et al. TP53: *TP53* disruption/aneuploidy. Spliceosome/chromatin: aberrations in spliceosome/chromatin genes. *JAK2* homo: homozygous *JAK2* mutation.

| **Supplemental Table 4. Type of resistance to hydroxyurea according to genomic classification in 61 patients with polycythemia vera** | | | | |
| --- | --- | --- | --- | --- |
|  | Phlebotomies  N=13 | Uncontrolled  Myeloproliferation  N=6 | Progressive  Splenomegaly  N=3 | Cytopenia  N=39 |
| *TP53* disruption | 2 (15) | 1 (17) | 1 (33) | 6 (15) |
| Chromatin/ spliceosome | 2 (15) | 2 (33) | 1 (33) | 18 (47) |
| Homozygous  *JAK2* mutation | 3 (23) | 3 (50) | 1 (33) | 10 (26) |
| Heterozygous  *JAK2* mutation | 5 (38) | 0 (0) | 0 (0) | 5 (13) |
| No known driver mutation | 1 (8) | 0 (0) | 0 (0) | 0(0) |

Results are expressed in number of patients (percentages). HU: hydroxyurea. Resistance to HU as per the ELN 2010 modified criteria, including: need for phlebotomy to keep hematocrit < 0.45 L/L after 3 months of the maximum tolerated dose (MTD) of HU; uncontrolled myeloproliferation (platelet count > 400 x 10^9^/L and WBC count > 10 x 10^9^/L after 3 months of at least 2 g/day or MTD of HU); failure to reduce massive splenomegaly after 3 months of MTD of HU; absolute neutrophil count < 1 x 10^9^/L or Hb level < 100 g/L or platelet count < 100 x 10^9^/L at the lowest dose of HU required to achieve a complete or partial response.
